# Impact of Central Obesity on Women with Polycystic Ovary Syndrome Undergoing *In Vitro* Fertilization

**DOI:** 10.1089/biores.2017.0040

**Published:** 2018-08-01

**Authors:** Yu Li, Haiyan Lin, Ping Pan, Dongzi Yang, Qingxue Zhang

**Affiliations:** Department of Gynecology and Obstetrics, Reproductive Medicine Center, Sun Yat-sen Memorial Hospital, Sun Yat-sen University, Guangzhou, China.

**Keywords:** central obesity, *in vitro* fertilization, polycystic ovary syndrome, pregnancy outcome

## Abstract

Central obesity (CO) is a defining characteristic of polycystic ovary syndrome (PCOS) and PCOS-induced disorders are likely to be exacerbated in the presence of CO. This study aims to evaluate the impact of CO on infertile women with PCOS undergoing *in vitro* fertilization (IVF).It is a retrospective and case–control study. One hundred eighty-eight infertile PCOS women undergoing IVF were divided into CO group (*n* = 70, waist circumference [WC] ≥80 cm) and noncentral obesity (NCO) group (*n* = 118, WC <80 cm). Baseline characteristics, parameters of ovarian stimulation and laboratory, and pregnancy outcomes were compared between two groups. After controlling for body mass index (BMI), WC positively correlated with fasting insulin (*r* = 0.210, *p* = 0.007), homeostatic model assessment for insulin resistance (*r* = 0.249, *p* = 0.006) and free androgen index (*r* = 0.249, *p* = 0.006). Compared with NCO group, CO group had significantly increased endocrine and metabolic disorders and needed significantly higher dose of gonadotropins, longer duration of ovarian stimulation (*p* < 0.05), but had significantly lower peak serum estradiol level (*p* < 0.01) and less oocytes retrieved (*p* = 0.032). CO group had significantly lower live birth and implantation rates (53.8% vs. 86.8%, *p* = 0.001; and 24.3% vs. 36.3%, *p* = 0.019, respectively) and higher early spontaneous miscarriage rate (38.5% vs. 7.5%, *p* = 0.002). For the multivariate analysis, by adjusting for age, BMI, insulin resistance, and hyperandrogenism (HA), CO was significantly independent risk factor for early miscarriage (adjusted relative ratio = 16.87, 95% confidence interval = 2.15–132.70, *p* = 0.007). CO is associated with insulin resistance, hyperinsulinemia, and HA independent of BMI and is associated with poor pregnancy outcome in infertile women with PCOS undergoing IVF.

## Introduction

Polycystic ovary syndrome (PCOS) is the most common and complicated endocrine disorder in women of reproductive age, with multiple endocrine and metabolic disorders. Subfertile women with PCOS will usually benefit from conventional treatments, such as lifestyle changes, laparoscopic ovarian drilling, or ovulation induction.^1^ However, when undergoing *in vitro* fertilization (IVF)/intracytoplasmic sperm injection (ICSI), PCOS women may suffer from high rates of cycle cancellations, spontaneous miscarriage, and decreased rate of fertilization.^2–4^

PCOS women represent a cohort of people with a higher prevalence of overweight and obesity compared with healthy women,^5^ although there is a wide variability in the estimates of obesity in PCOS women across different countries and ethnicities.^6^ The prevalence ranges from 66% to 80% in PCOS women from the Western populations^7,8^ and about 23% in Chinese PCOS women.^9^

It is found that overweight and obesity are closely related to the severity of endocrine disorders, such as insulin resistance, impaired glucose tolerance, and other metabolic abnormalities in PCOS women.^10–12^ Furthermore, it is also important to emphasize that visceral, rather than subcutaneous, fat, also named central obesity (CO), is a defining characteristic of PCOS.^13^ CO can induce local and systemic oxidative stress in PCOS patients, which suggest that PCOS-induced disorders are likely to be exacerbated in the presence of CO.^14^

The present study aims to evaluate whether CO is associated with ovarian stimulation and pregnancy outcome in Chinese infertile PCOS women undergoing IVF/ICSI.

## Materials and Methods

### Study population

This was a retrospective study. From January 2009 to December 2010, PCOS women who accepted their first fresh IVF/ICSI cycles at the IVF center of Sun Yat-sen Memorial Hospital, Sun Yat-sen University were included. This study was approved by the local Medical Ethics Committee.

The PCOS patients were diagnosed according to the 2003 Rotterdam diagnostic criteria.^15^ Patients were excluded if they had uterine malformation, untreated intrauterine lesions, and previous ovarian surgery. Women with waist circumference (WC) more than 80 cm were defined as CO based on the 2005 International Diabetes Foundation consensus (http://idf.org/home). Patients were divided into CO group and noncentral obesity (NCO) group.

The phenotypes in PCOS women were divided according to previous studies.^16,17^ Following these studies, PCOS features were defined hyperandrogenism (HA), oligoanovulatory ovarian dysfunction (OAD), and polycystic ovarian morphology (PCOM). Then four different PCOS phenotypes were identified: phenotype A or complete phenotype (HA, OAD, and PCOM), phenotype B or non-PCOM phenotype (HA and OAD without PCOM), phenotype C or ovulatory PCOS phenotype (HA and PCOM with ovulatory cycles), phenotype D or normoandrogenic PCOS (OAD and PCOM without HA).

### Clinical and biochemical measurements

Data on characteristic features, ultrasonographic, and laboratory variables were collected. The height, weight, WC, and hip circumference of a patient were measured according to World Health Organization recommendation.^18^ Transvaginal ultrasonography was performed on day 3–5 of menstrual cycle or after a progestin-induced withdrawal bleeding to exclude any pelvic pathology and to determine the antral follicle count (AFC).

Serum hormone levels (follicle-stimulating hormone [FSH], luteinizing hormone [LH], estradiol [E_2_], total testosterone [TT], androstenedione, free testosterone [FT], dehydroepiandrosterone sulfate [DHEA-S], estrone, sex hormone-binding globulin [SHBG], and 17α-hydroxyprogesterone) on day 3–5 of menstrual cycle or after a progestin-induced withdrawal bleeding and other serum biochemical evaluations (cholesterol, triglycerides [TG], high-density lipoprotein-cholesterol [HDL-C], low-density lipoprotein [LDL-C] levels, plasma glucose, and insulin) were measured.

Impaired fasting glucose (IFG), impaired glucose tolerance (IGT), and type 2 diabetes mellitus (DM2) were diagnosed according to American Diabetes Association 2007 Standards.^19^ The homeostatic model assessment for insulin resistance (HOMA-IR) and free androgen index (FAI) were calculated as following: (fasting plasma insulin [mIU/L] × fasting plasma glucose [mmol/l])/22.5, and (100 × TT [nmol/L])/SHBG [nmol/L]), respectively. Insulin resistance was defined as HOMA-IR >2.14 according to our previous study.^20^

### IVF/ICSI and embryo transfer procedures

All PCOS patients were pretreated according to the consensus on PCOS patients' treatment.^21^ When patients were defined as insulin resistant, IFG, or IGT, metformin was administrated for 1–3 month. Standard controlled ovarian stimulation protocol was performed as follows.^22^ All patients were treated with oral contraceptive pills (Yasmin, Scherring, Germany) from cycle days 3 or days 3 after a progestin-induced withdrawal bleeding.

Then long-acting triptorelin acetate (Diphereline, Ipsen, France) was given at the 17th–19th day with a single dose of 1.25 mg subcutaneously. Fourteen days after triptorelin acetate was given, when the endometrium with the thickness of ≤5 mm and suppressed ovaries (no antral follicles ≥10 mm) were detected by ultrasound scan and serum estradiol levels ≤50 pg/mL and LH levels ≤5 IU/L were confirmed, ovarian stimulation was started. Around 112.5–225 IU/day recombinant FSH (Gonal-F; Merck Serono, Germany) was administered according to the patient's age, AFC, and basal FSH level. If necessary, the dosage of recombinant FSH was adjusted or 75–150 IU/day recombinant LH (Luveris; Merck Serono, Germany) was added according to ovarian response.

Urinary human chorionic gonadotropin (hCG; Lizhu, China) was given for triggering when at least two follicles had reached a diameter of ≥18 mm or three follicles had reached a diameter of ≥17 mm. Around 4000–10,000 IU of hCG was administrated depending on follicular numbers, peak E_2_ level, and body mass index (BMI). Transvaginal oocyte retrieval was scheduled 36–37 h after the hCG injection.

Then, standard laboratory procedures for conventional IVF/ICSI were followed.^23^ Evaluation of the quality of embryos was performed on day 3 according to “embryo grading”.^24^ Embryos of modified Hu's grades 1 or 2 were considered of high quality (7–9 cell; blastomeres of equal size; 0–20% cytoplasmic fragments). When available, two embryos were transferred in young women (<35 years) and three embryos were transferred in older women (≥35 years). Embryo transfers (ET) were performed under ultrasound guidance with a full bladder using a Wallace catheter (Wallace Ltd, Colchester, England). Luteal phase support was sustained with natural progesterone in oil 60 mg intramuscular injection daily from the day of oocyte retrieval.

### Pregnancy outcomes

Biochemical pregnancy was defined as elevated serum β-hCG (≥25 IU/L) 14 days after ET. Clinical pregnancy was defined as the presence of gestational sac(s) by ultrasonography 4–5 weeks after ET.

### Statistical analysis

Statistical analysis of the data was performed using SPSS version 21.0 (SPSS, Inc., Chicago, IL). Continuous variables were compared using Student's *t*-test or analysis of variance for parametric data and Mann–Whitney test or Kruskal–Wallis test for nonparametric data. Categorical data were compared using Chi-squared test. Pearson partial correlation was used for correlation analysis between two continuous variables after adjusting some confounding factor.

We estimated the crude relative ratio (RR) and 95% confidence interval (CI) for the relation between early miscarriage and other confounders, such as age, BMI, CO, fasting glucose insulin, and FAI. For the multivariate analysis, we adjusted these confounders. The RR was calculated using a median unbiased estimator for binary data in an unconditional logistic regression model. All tests were considered statistically significant at *p* < 0.05.

## Results

### Baseline characteristics

A total of 188 women with PCOS met the eligibility criteria. There were 70 and 118 women within the CO and NCO groups, respectively.

The baseline characteristics of the subjects are shown in Table 1. When comparing CO with NCO group, BMI, WC, hip circumference, waist–hip ratio, FAI, fasting glucose, fasting insulin (FIN), HOMA-IR, 2-h glucose and insulin in oral glucose tolerance test, serum TG, and the occurrence rates of IR, IFG, IGT were significantly higher, whereas serum SHBG and HDL-C was significantly lower (*p* < 0.05). There was no significant difference in age, duration of infertility, baseline serum hormones (FSH, LH, TT, FT, DHEA-S), Serum TC, LDL-C, bilateral volumes of ovaries, AFC, and rates of primary infertility and DM2 between the two groups (*p* > 0.05). In addition, the PCOS phenotypes were not significant between the two groups (*p* > 0.05).

**Table 1.** **Baseline Characteristics in Polycystic Ovary Syndrome Women With or Without Central Obesity**

	Central obesity (*n* = 70)	Noncentral obesity (*n* = 118)
No. of cycles	70	118
Age (years)	30.2 ± 3.5	29.5 ± 3.5
Duration of infertility (years)	5.38 ± 3.28	4.76 ± 3.15
Primary infertility (*n*, %)	42 (60.0)	72 (61.0)
Cause of infertility (*n*)		
PCOS	20	28
PCOS and tubal factor	38	64
PCOS and male factor	10	20
PCOS and endometriosis	2	6
Times of cycle	1.21 ± 0.41	1.16 ± 0.37
PCOS phenotypes		
HA, OAD, and PCOM	39 (55.7)	53 (44.9)
HA and OAD without PCOM	13 (18.6)	17 (14.4)
HA and PCOM with ovulatory cycles	4 (5.7)	16 (13.6)
OAD and PCOM without HA	14 (20.0)	32 (27.1)
BMI (kg/m^2^)	24.9 ± 2.2^*^	20.4 ± 2.4^*^
Waist circumference (cm)	86.1 ± 6.2^*^	71.0 ± 4.4^*^
Hip circumference (cm)	97.5 ± 6.4^*^	89.3 ± 4.2^*^
Waist–hip ratio	0.88 ± 0.06^*^	0.80 ± 0.05^*^
Basal FSH (IU/L)	7.21 ± 2.82	7.32 ± 2.30
Basal LH (IU/L)	7.90 ± 5.13	8.09 ± 5.32
Basal TT (nmol/L)	2.03 ± 0.72	2.14 ± 0.87
FT (pg/mL)	3.92 ± 2.37	3.11 ± 2.35
FAI	5.73 ± 4.73^*^	3.17 ± 2.47^*^
DHEA-S (ng/mL)	2231.4 ± 983.6	2200.4 ± 1077.1
SHBG (nmol/L)	57.8 ± 44.8^*^	103.4 ± 85.1^*^
Serum fasting glucose (mmol/L)	5.35 ± 0.78^**^	5.08 ± 0.44^**^
Serum fasting insulin (mU/L)	11.63 ± 7.17^*^	6.34 ± 3.63^*^
HOMA-IR	2.87 ± 2.44^*^	1.44 ± 0.84^*^
Insulin resistance (*n*, %)	45 (64.3)^*^	20 (16.9) ^*^
2-h glucose (mmol/L)	8.38 ± 3.03^*^	6.54 ± 2.14^*^
2-h insulin (mU/mL)	111.98 ± 68.36^*^	64.26 ± 47.65^*^
IFG (*n*, %)	17 (24.3)^**^	14 (11.9)^**^
IGT (*n*, %)	17 (24.3)^**^	13 (11.0)^**^
Type II DM (*n*, %)	8 (11.4)	4 (0.3)
CHOL (mmol/L)	5.11 ± 0.50	4.83 ± 0.90
TG (mmol/L)	2.70 ± 2.30^*^	1.25 ± 0.65^*^
HDL-C (mmol/L)	1.37 ± 1.35^**^	1.52 ± 0.27^**^
LDL-C (mmol/L)	3.10 ± 0.68	2.80 ± 0.85
Volume of left ovary (mL)	7.68 ± 5.92	7.23 ± 3.17
Volume of right ovary (mL)	7.46 ± 6.13	7.82 ± 4.34
AFC	25.8 ± 9.1	25.8 ± 8.5

Values are expressed as mean ± SD or numbers (percentages).

^*^ Significant differences between two groups, *p* < 0.01.

^**^ Significant differences between two groups, *p* < 0.05.

PCOS, polycystic ovary syndrome; PCOM, polycystic ovarian morphology; HA, hyperandrogenism; OAD, oligoanovulatory ovarian dysfunction; BMI, body mass index; FSH, follicle-stimulating hormone; LH, luteinizing hormone; TT, total testosterone; FT, free testosterone; FAI, free androgen index; DHEA-S, dehydroepiandrosterone sulfate; SHBG, sex hormone-binding globulin; HOMA-IR, homeostatic model assessment for insulin resistance; IFG, impaired fasting glucose; IGT, impaired glucose tolerance; DM, diabetes mellitus; CHOL, cholesterol; TG, triglycerides; HDL-C, high-density lipoprotein-cholesterol; LDL-C, low-density lipoprotein-cholesterol; AFC, antral follicle count; SD, standard deviation.

After controlling for BMI, WC positively correlated with FIN (*r* = 0.210, *p* = 0.007), HOMA-IR (*r* = 0.249, *p* = 0.006), and FAI (*r* = 0.249, *p* = 0.006).

### Parameters during IVF/ICSI

The detailed parameters during IVF/ICSI treatment are shown in Table 2. One cycle was cancelled in CO group, whereas three in NCO group due to poor ovarian response. Compared with NCO group, CO group needed significantly higher dose of gonadotropin, longer duration of ovarian stimulation, higher dose of hCG for trigger(*p* < 0.05), but had significantly lower peak serum estradiol level, less mature follicles on trigger day, and less oocytes retrieved (*p* < 0.05). There were no significant differences in endometrial thickness, cleavage rate, number of frozen embryos, percentage of embryos available, number of high-quality embryos, and rates of completed cycles, ICSI cycles, and fertilization rate between the two groups.

**Table 2.** **Parameters During *In Vitro* Fertilization in Polycystic Ovary Syndrome Women With or Without Central Obesity**

	Central obesity (*n* = 70)	Noncentral obesity (*n* = 118)
Cycle cancellation for poor response (*n*, %)	1 (1.4)	3 (2.5)
Total dose of gonadotropin (IU)	2014.8 ± 825.8^*^	1491.2 ± 558.9^*^
Duration of gonadotropin stimulation (days)	12.5 ± 4.0^*^	10.7 ± 3.0^*^
Peak E_2_ (pg/mL)	2320.4 ± 1303.6^*^	3256.3 ± 1456.0^*^
Endometrial thickness (mm)	11.7 ± 3.0	11.5 ± 2.8
Dose of hCG (IU)	6869.6 ± 1971.6^**^	6134.8 ± 1652.1^**^
No. of mature follicles	9.6 ± 4.6^**^	11.5 ± 5.2^**^
No. of completed cycles (*n*, %)	69 (98.6)	115 (97.5)
No. of ICSI cycles (*n*, %)	12 (17.1)	23 (19.5)
No. of oocytes retrieved	11.6 ± 5.7^**^	13.8 ± 7.3^**^
Fertilization rate (%)	0.64 ± 0.24	0.61 ± 0.21
Cleavage rate (%)	0.96 ± 0.16	0.97 ± 0.14
No. of frozen embryos	4.72 ± 4.50	5.98 ± 5.35
Percentage of embryos available	0.74 ± 0.23	0.73 ± 0.22
No. of high quality embryos	3.67 ± 3.14	4.03 ± 3.96

Values are expressed as mean ± SD or numbers (percentages).

^*^ Significant differences between two groups, *p* < 0.01.

^**^ Significant differences between two groups, *p* < 0.05.

E_2_, estradiol; hCG, human chorionic gonadotropin; ICSI, intracytoplasmic sperm injection.

### Pregnancy outcomes

CO group had significantly lower rates of implantation and live birth (24.3% vs. 36.3%, *p* = 0.019; and 53.8% vs. 86.8%, *p* = 0.001, respectively) and higher rate of early spontaneous miscarriage (38.5% vs. 7.5%, *p* = 0.002), compared with the NCO group (Table 3). Two groups showed similar rates of moderate ovarian hyper-stimulation syndrome (OHSS), biochemical, clinical, multiple and ectopic pregnancy, and number of transferred embryos.

**Table 3.** **Pregnancy Outcomes in Polycystic Ovary Syndrome Women With or Without Central Obesity**

	Central obesity (*n* = 70)	Noncentral obesity (*n* = 118)
Moderate OHSS (*n*, %)	4 (5.8)	10 (8.7)
Embryo transfer cancellation for risk of OHSS (*n*, %)	5 (7.2)	17 (13.8)
Embryo transfer cycles (*n*, %)	64 (91.4)	96 (81.4)
No. of embryos transferred	2.14 ± 0.53	2.13 ± 0.42
Biochemical pregnancy (*n*, %)	28 (43.8)	56 (58.3)
Clinical pregnancy (%)	26/64 (40.6)	53/96 (55.2)
Implantation (%)	34/140 (24.3)^*^	74/204 (36.3)^*^
Multiple pregnancies (%)	8/26 (30.8)	22/53 (41.5)
Early miscarriage (%)	10/26 (38.5)^**^	4/53 (7.5)^**^
Ectopic pregnancy (%)	0/26 (0)	1/53 (1.9)
Live birth (%)	14/26 (53.8)^**^	46/53 (86.8)^**^

Values are expressed as mean ± SD or numbers (percentages).

^*^ Significant differences between two groups, *p* < 0.05.

^**^ Significant differences between two groups, *p* < 0.01.

Biochemical pregnancy rate: biochemical pregnancy cycles/embryo transfer cycles; clinical pregnancy rate: clinical pregnancy cycles/embryo transfer cycles; implantation rate: no. of implantation gestational sac/no. of total embryos transferred; multiple pregnancy rates: multiple pregnancy cycles/clinical pregnancy cycles; early miscarriage rate: early miscarriage cycles/clinical pregnancy cycles; ectopic pregnancy rate: ectopic pregnancy cycles/clinical pregnancy cycles; live birth rate: live birth cycles/clinical pregnancy cycles.

OHSS, ovarian hyper-stimulation syndrome.

Multivariate analysis for early miscarriage showed that when compared with NCO, the crude RR for CO was 7.50 (95% CI = 2.06–27.25, *p* = 0.002) and remained statistically significant (adjusted RR = 16.87, 95% CI = 2.15–132.70, *p* = 0.007) after adjusting for age, BMI, insulin resistance, and HA (Table 4). In addition, the RR for age was also very strong (crude RR = 25.20, 95% CI = 2.55–249.00, *p* = 0.006) for the early miscarriage and similar after adjustment (adjusted RR = 43.39, 95% CI = 2.15–692.53, *p* = 0.008). While the association with BMI, insulin resistance, and HA was not significant (*p* < 0.05).

**Table 4.** **Crude and Adjusted Relative Ratios for Early Miscarriage**

	Crude RR (95% CI)	*p*	Adjusted RR^a^ (95% CI)	*p*
Age (years)				
>35 vs. ≤35	25.20 (2.55–249.00)	0.006	43.39 (2.15–692.53)	0.008
BMI (kg/m^2^)				
<18.5 vs. >24	0.67 (0.06–7.23)	0.739	2.96 (0.05–174.47)	0.602
18.5–23.9 vs. >24	0.42 (0.12–1.45)	0.172	1.57 (0.22–11.32)	0.653
Central obesity				
Yes vs. No	7.50 (2.06–27.25)	0.002	16.87 (2.15–132.70)	0.007
Insulin resistance				
Yes vs. No	1.95 (0.60–6.34)	0.267	1.02 (0.21–4.95)	0.983
Hyperandrogenism				
Yes vs. No	1.67 (0.42–6.64)	0.469	1.35 (0.24–7.59)	0.732

^a^ Adjusted for age, BMI, central obesity, insulin resistance and hyperandrogenism.

RR, relative ratio; CI, confidence interval.

## Discussion

PCOS is the most common and complicated endocrine dysfunction in women of childbearing age. A majority of PCOS patients are obese or have a normal BMI, but are metabolically obese with WC more than 80 cm. Pregnancy achievement and maintenance is adversely affected by obesity, overweight, or elevated BMI. Huang et al. reported that obese PCOS patients obtained lower clinical pregnancy and live birth rates.^25^ However, WC may be a good indicator to identify insulin resistance and metabolic syndrome, especially among population of BMI ≤30 (kg/m^2^).^26,27^ WCs seem to help identify those at increased health risk within the normal weight, overweight, and class I obese BMI categories.^26^

This study was to evaluate the effects of CO on the outcomes of IVF/ICSI in women with PCOS. Among these patients in this study, 98.9% women had BMI ≤30 kg/m^2^; CO was associated with endocrinal and metabolic disorders in women with PCOS and was significantly associated with insulin resistance, hyperinsulinemia, and HA independent of BMI. This suggested that WC could be a better marker than BMI for Chinese women, which is in accordance with a study conducted by Stepto et al.^28^ Therefore, WC could be a good and simple indicator for distinguishing patients with different metabolic status in PCOS women, to CO and NCO groups.

Previous studies had shown the PCOS phenotypes played an important role in the variability of pregnancy complication^16^ and oocyte competence.^17^ Therefore, in this study, patients were subclassified according to the four phenotypes, and there were no significant differences between the two groups.

Obese PCOS patients needed more dose of gonadotropins, had less mature follicles on hCG day, and less oocytes retrieved after COS.^29–31^ Altered pharmacokinetics of gonadotropin and high intrafollicular leptin concentration may be related to gonadotropin resistance in obese PCOS women.^30^ Patients with gonadotropin resistance might have increased the rate of cycle cancellations due to poor ovary responses in common population. However, the condition of increased cycle cancellations did not appear in our CO group. It may be because more AFC was found in PCOS patients, which can recruit more follicles after adjusting the dose of gonadotropin during ovarian stimulation. CO patients accepted higher dose of gonadotropin, which may increase follicle recruitment and oocytes retrieved.

The present study indicated that PCOS women in CO group had decreased clinical pregnancy rate, significantly higher early spontaneous miscarriage rate, and lower live birth rate compared with the NCO group. Insulin resistance may be responsible for this condition.^32,33^ Moreover, in CO group, the number of high-quality embryos, and rate of biochemical pregnancy were lower than NCO group, although it was not significant. In multivariate analysis we could see that CO was a significantly independent risk factor for early miscarriage. It remained significant after adjusting for insulin resistance, which indicated that PCOS infertile women with CO in China were at a greater risk in early spontaneous miscarriage.

Possible mechanism of poor pregnancy outcome in PCOS women with CO is that metabolic disturbance such as insulin resistance result in abnormalities during folliculogenesis, follicular growth, oocyte meiotic maturation processes, and uterine receptivity, through circulating endocrine and local paracrine/autocrine mechanisms.^34–36^ With regard to incidence of moderate OHSS and freeze-all cycles due to the risk of OHSS, the NCO group showed higher rates. It can be explained that lean PCOS patients are a high-risk group of OHSS in itself.^37^

The special strength of this study was that CO could be a better indicator to identify the comprehensive endocrinal and metabolic disorders and poor pregnancy outcome undergoing IVF treatment among Chinese women with PCOS, who have much higher proportion of normal weight, overweight, and class I obese BMI categories than western population.

The main limitation of this study is the relatively small sample size and retrospective nature. Further studies in larger populations are needed to come up with more supporting data. Furthermore, because of differences in ethnicity, effects of the CO on PCOS women could account for some conflicting results. However, to our knowledge, this study is the first to identify the association of CO and pregnancy outcome in PCOS women undergoing IVF/ICSI.

In summary, PCOS patients with CO have severe insulin resistance and metabolic disorders independent of BMI. When undergoing IVF, they need more dose of gonadotrophin and longer duration of ovarian stimulation, but have lower number of retrieved oocytes. PCOS patients with CO have higher early miscarriage rate, reduced implantation, and live birth rates after IVF/ICSI.

## Details of Ethics Approval

This study was approved by the Ethics Committee of Sun Yat-sen Memorial Hospital, Sun Yat-sen University (No. 201408).

## Abbreviations Used

AFC: antral follicle count
BMI: body mass index
CHOL: cholesterol
CI: confidence interval
CO: central obesity
DHEA-S: dehydroepiandrosterone sulfate
DM: diabetes mellitus
DM2: type 2 diabetes mellitus
E2: estradiol
ET: embryo transfers
FAI: free androgen index
FIN: fasting insulin
FSH: follicle-stimulating hormone
FT: free testosterone
HA: hyperandrogenism
hCG: human chorionic gonadotropin
HDL-C: high-density lipoprotein-cholesterol
HOMA-IR: homeostatic model assessment for insulin resistance
IFG: impaired fasting glucose
IGT: impaired glucose tolerance
IVF: *in vitro* fertilization
LDL-C: low-density lipoprotein
LH: luteinizing hormone
NCO: noncentral obesity
OAD: oligoanovulatory ovarian dysfunction
PCOM: polycystic ovarian morphology
PCOS: polycystic ovary syndrome
RR: relative ratio
SD: standard deviation
SHBG: sex hormone-binding globulin
TG: triglycerides
TT: total testosterone
WC: waist circumference

## References

[1] ESHRE Capri Workshop Group. ECW. Health and fertility in World Health Organization group 2 anovulatory women. Hum Reprod Update. 2012;18:586–599 22611175 10.1093/humupd/dms019

[2] Heijnen EM, Eijkemans MJ, Hughes EG, et al. A meta-analysis of outcomes of conventional IVF in women with polycystic ovary syndrome. Hum Reprod Update. 2006;12:13–21 16123051 10.1093/humupd/dmi036

[3] Boomsma CM, Fauser BC, Macklon NS Pregnancy complications in women with polycystic ovary syndrome. Semin Reprod Med. 2008;26:72–84 18181085 10.1055/s-2007-992927

[4] Okohue JE, Onuh SO, Ikimalo JI Comparison of IVF/ICSI outcome in patients with polycystic ovarian syndrome or tubal factor infertility. Niger J Clin Pract. 2013;16:207–210 23563463 10.4103/1119-3077.110164

[5] Lim SS, Davies MJ, Norman RJ, et al. Overweight, obesity and central obesity in women with polycystic ovary syndrome: a systematic review and meta-analysis. Hum Reprod Update. 2012;18:618–637 22767467 10.1093/humupd/dms030

[6] Fauser BC, Tarlatzis BC, Rebar RW, et al. Consensus on women's health aspects of polycystic ovary syndrome (PCOS): the Amsterdam ESHRE/ASRM-Sponsored 3rd PCOS Consensus Workshop Group. Fertil Steril. 2012;97:28.e25–38.e25 22153789 10.1016/j.fertnstert.2011.09.024

[7] Azziz R, Sanchez LA, Knochenhauer ES, et al. Androgen excess in women: experience with over 1000 consecutive patients. J Clin Endocrinol Metab. 2004;89:453–462 14764747 10.1210/jc.2003-031122

[8] Hahn S, Tan S, Sack S, et al. Prevalence of the metabolic syndrome in German women with polycystic ovary syndrome. Exp Clin Endocrinol Diabetes. 2007;115:130–135 17318774 10.1055/s-2007-967093

[9] Ni RM, Mo Y, Chen X, et al. Low prevalence of the metabolic syndrome but high occurrence of various metabolic disorders in Chinese women with polycystic ovary syndrome. Eur J Endocrinol. 2009;161:411–418 19542239 10.1530/EJE-09-0298

[10] Vrbikova J, Hainer V Obesity and polycystic ovary syndrome. Obes Facts. 2009;2:26–35 20054201 10.1159/000194971 PMC6444522

[11] Baldani DP, Skrgatic L, Ougouag R Polycystic ovary syndrome: important under-recognised cardiometabolic risk factor in reproductive-age women. Int J Endocrinol. 2015;2015:786362 26124830 10.1155/2015/786362 PMC4466395

[12] Cascella T, Palomba S, De Sio I, et al. Visceral fat is associated with cardiovascular risk in women with polycystic ovary syndrome. Hum Reprod. 2008;23:153–159 18024952 10.1093/humrep/dem356

[13] Ross R, Janssen I, Dawson J, et al. Exercise-induced reduction in obesity and insulin resistance in women: a randomized controlled trial. Obes Res. 2004;12:789–798 15166299 10.1038/oby.2004.95

[14] Nasiri N, Moini A, Eftekhari-Yazdi P, et al. Abdominal obesity can induce both systemic and follicular fluid oxidative stress independent from polycystic ovary syndrome. Eur J Obstet Gynecol Reprod Biol. 2015;184:112–116 25498475 10.1016/j.ejogrb.2014.11.008

[15] Rotterdam ESHRE/ASRM-Sponsored PCOS Consensus Workshop Group. Revised 2003 consensus on diagnostic criteria and long-term health risks related to polycystic ovary syndrome (PCOS). Hum Reprod. 2004;19:41–47 10.1093/humrep/deh098 14688154

[16] Palomba S, de Wilde MA, Falbo A, et al. Pregnancy complications in women with polycystic ovary syndrome. Hum Reprod Update. 2015;21:575–592 26117684 10.1093/humupd/dmv029

[17] Palomba S, Daolio J, La Sala GB Oocyte competence in women with polycystic ovary syndrome. Trends Endocrinol Metab. 2017;28:186–198 27988256 10.1016/j.tem.2016.11.008

[18] World Health Organization, Nutrition Unit, EUR ICP/NUT. M*easuring Obesity: Classification and Distribution of Anthropometric Data* WHO: Copenhagen, Denmark; p. 125; 1989

[19] American Diabetes Association. Standards of medical care in diabetes—2007. Diabetes Care. 2007;30(Suppl 1):S4–S41 17192377 10.2337/dc07-S004

[20] Chen X, Yang D, Li L, et al. Abnormal glucose tolerance in Chinese women with polycystic ovary syndrome. Hum Reprod. 2006;21:2027–2032 16684838 10.1093/humrep/del142

[21] Group TEA-SPCW. Consensus on infertility treatment related to polycystic ovary syndrome. Fertil Steril. 2008;89:505–522 18243179 10.1016/j.fertnstert.2007.09.041

[22] Li Y, Yang D, Zhang Q Clinical outcome of one-third-dose depot triptorelin is the same as half-dose depot triptorelin in the long protocol of controlled ovarian stimulation. J Hum Reprod Sci. 2012;5:14–19 22870009 10.4103/0974-1208.97785 PMC3409913

[23] Magli MC, Van den Abbeel E, Lundin K, et al. Revised guidelines for good practice in IVF laboratories. Hum Reprod. 2008;23:1253–1262 18375408 10.1093/humrep/den068

[24] Hu Y, Maxson WS, Hoffman DI, et al. Maximizing pregnancy rates and limiting higher-order multiple conceptions by determining the optimal number of embryos to transfer based on quality. Fertil Steril. 1998;69:650–657 9548153 10.1016/s0015-0282(98)00024-7

[25] Huang K, Liao X, Dong X, et al. Effect of overweight/obesity on IVF-ET outcomes in chinese patients with polycystic ovary syndrome. Int J Clin Exp Med. 2014;7:5872–5876 25664123 PMC4307570

[26] Ascaso JF, Romero P, Real JT, et al. Abdominal obesity, insulin resistance, and metabolic syndrome in a southern European population. Eur J Intern Med. 2003;14:101–106 12719027 10.1016/S0953-6205(03)00022-0

[27] Janssen I, Katzmarzyk PT, Ross R Body mass index, waist circumference, and health risk: evidence in support of current National Institutes of Health guidelines. Arch Intern Med. 2002;162:2074–2079 12374515 10.1001/archinte.162.18.2074

[28] Stepto NK, Cassar S, Joham AE, et al. Women with polycystic ovary syndrome have intrinsic insulin resistance on euglycaemic-hyperinsulaemic clamp. Hum Reprod. 2013;28:777–784 23315061 10.1093/humrep/des463

[29] McCormick B, Thomas M, Maxwell R, et al. Effects of polycystic ovarian syndrome on in vitro fertilization-embryo transfer outcomes are influenced by body mass index. Fertil Steril. 2008;90:2304–2309 18191852 10.1016/j.fertnstert.2007.10.077

[30] Fedorcsak P, Dale PO, Storeng R, et al. The impact of obesity and insulin resistance on the outcome of IVF or ICSI in women with polycystic ovarian syndrome. Hum Reprod. 2001;16:1086–1091 11387273 10.1093/humrep/16.6.1086

[31] Jungheim ES, Lanzendorf SE, Odem RR, et al. Morbid obesity is associated with lower clinical pregnancy rates after in vitro fertilization in women with polycystic ovary syndrome. Fertil Steril. 2009;92:256–261 18692801 10.1016/j.fertnstert.2008.04.063 PMC3746795

[32] Hong Y, Xie QX, Chen CY, et al. Insulin resistance in first-trimester pregnant women with pre-pregnant glucose tolerance and history of recurrent spontaneous abortion. J Biol Regul Homeost Agents. 2013;27:225–231 23489701

[33] Banu J, Fatima P, Sultana P, et al. Association of infertile patients having polycystic ovarian syndrome with recurrent miscarriage. Mymensingh Med J. 2014;23:770–773 25481599

[34] Qiao J, Feng HL Extra- and intra-ovarian factors in polycystic ovary syndrome: impact on oocyte maturation and embryo developmental competence. Hum Reprod Update. 2011;17:17–33 20639519 10.1093/humupd/dmq032 PMC3001338

[35] Dumesic DA, Abbott DH Implications of polycystic ovary syndrome on oocyte development. Semin Reprod Med. 2008;26:53–61 18181083 10.1055/s-2007-992925 PMC2655636

[36] Wood JR, Dumesic DA, Abbott DH, et al. Molecular abnormalities in oocytes from women with polycystic ovary syndrome revealed by microarray analysis. J Clin Endocrinol Metab. 2007;92:705–713 17148555 10.1210/jc.2006-2123

[37] Aboulghar M Prevention of OHSS. Symposium: update on prediction and management of ohss. Reprod Biomed Online. 2009;19:33–42 10.1016/s1472-6483(10)60043-0 19573288

